# Characterisation of the static offset in the travelling wave in the cochlear basal turn

**DOI:** 10.1007/s00424-020-02373-6

**Published:** 2020-04-22

**Authors:** Takeru Ota, Fumiaki Nin, Samuel Choi, Shogo Muramatsu, Seishiro Sawamura, Genki Ogata, Mitsuo P. Sato, Katsumi Doi, Kentaro Doi, Tetsuro Tsuji, Satoyuki Kawano, Tobias Reichenbach, Hiroshi Hibino

**Affiliations:** 1 Department of Molecular Physiology, Niigata University School of Medicine, 1-757 Asahimachi-dori, Chuo-ku, Niigata, 951-8510 Japan; 2 AMED-CREST, AMED, Niigata, 951-8510 Japan; 3 Department of Electrical and Electronics Engineering, Niigata University, Niigata, 950-2181 Japan; 4 Department of Otolaryngology, Kindai University Faculty of Medicine, Osaka, 589-8511 Japan; 5 Department of Mechanical Science and Bioengineering, Graduate School of Engineering Science, Osaka University, Osaka, 560-8531 Japan; 6 Present Address: Department of Advanced Mathematical Sciences, Graduate School of Informatics, Kyoto University, Kyoto, 606-8501 Japan; 7 Department of Bioengineering, Imperial College London, London, SW7 2AZ UK

**Keywords:** Offset, Outer hair cells, Somatic motility, Travelling wave, Vibration

## Abstract

In mammals, audition is triggered by travelling waves that are evoked by acoustic stimuli in the cochlear partition, a structure containing sensory hair cells and a basilar membrane. When the cochlea is stimulated by a pure tone of low frequency, a static offset occurs in the vibration in the apical turn. In the high-frequency region at the cochlear base, multi-tone stimuli induce a quadratic distortion product in the vibrations that suggests the presence of an offset. However, vibrations below 100 Hz, including a static offset, have not been directly measured there. We therefore constructed an interferometer for detecting motion at low frequencies including 0 Hz. We applied the interferometer to record vibrations from the cochlear base of guinea pigs in response to pure tones. When the animals were exposed to sound at an intensity of 70 dB or higher, we recorded a static offset of the sinusoidally vibrating cochlear partition by more than 1 nm towards the scala vestibuli. The offset’s magnitude grew monotonically as the stimuli intensified. When stimulus frequency was varied, the response peaked around the best frequency, the frequency that maximised the vibration amplitude at threshold sound pressure. These characteristics are consistent with those found in the low-frequency region and are therefore likely common across the cochlea. The offset diminished markedly when the somatic motility of mechanosensitive outer hair cells, the force-generating machinery that amplifies the sinusoidal vibrations, was pharmacologically blocked. Therefore, the partition offset appears to be linked to the electromotile contraction of outer hair cells.

**Electronic supplementary material:**

The online version of this article (10.1007/s00424-020-02373-6) contains supplementary material, which is available to authorized users.

## Introduction

**Fig. 1:**
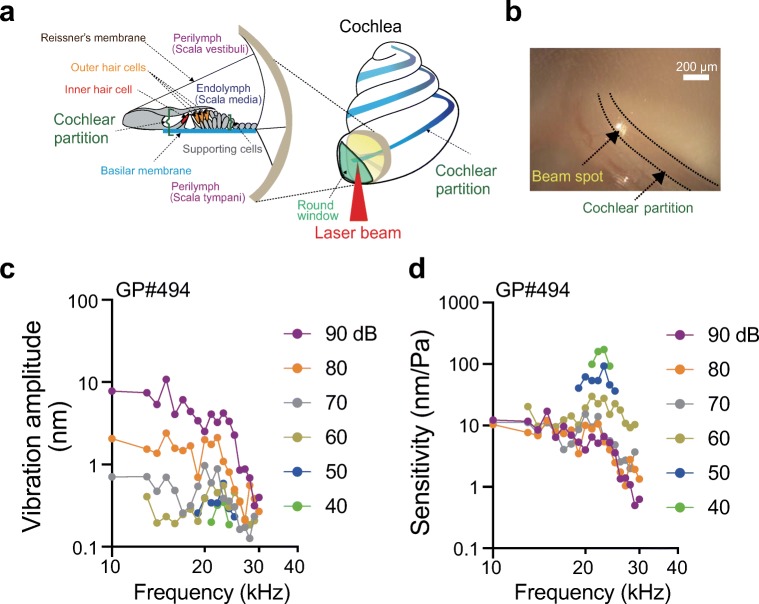
Preparation of the cochlea and evaluation of the procedure. **a** Structure of the cochlea (right panel) and its cross-section (left panel). A laser beam directly irradiated a spot of the cochlear partition in the basal turn through the round window membrane. **b** The position of interferometric measurement. This experimental view of the cochlear basal turn in a guinea pig was obtained under a microscope equipped with our SPM vibrometer. As shown in this panel, the cochlear partition was detected as a shadowed belt (highlighted by two dotted lines), and the beam was focused around the centre of this landmark. **c**, **d** Tuning curves. Guinea pig 494 (GP#494) was exposed to stimuli featuring a pair of different frequencies (10 and 13–30 kHz spaced by 1 kHz) and sound pressure (40–90 dB SPL; 10 dB steps), while the responses of the cochlear partition at the basal turn were monitored by the modified SPM interferometer. In panels **c** and **d**, the vibration amplitude and sensitivity—which was obtained by dividing the amplitude by sound pressure—are plotted against the stimulus frequency, respectively. In this preparation, the best frequency (BF) was 23 kHz. See the main text for the definition of the BF and Supplementary Text in Online Resource 3 for the detailed description of the results

In the mammalian hearing process, mechanical stimuli originating from a sound propagate through the middle ear and cause oscillatory pressure changes in the cochlea. The vibration elicits a wave that travels apically along the cochlear partition, a structure comprising the basilar membrane and overlying sensory hair cells and supporting cells [15, 50] (Fig. 1a). For a high-frequency stimulus, this travelling wave peaks near the cochlear base, whereas the wave reaches more apical positions at lower frequencies [50]. There are two types of hair cell, inner and outer; their cell bodies are bathed in perilymph, whereas their apical surfaces, from which the mechanosensitive hair bundles project, are exposed to a K^+^-rich extracellular solution termed endolymph. The hair bundle vibration that results from motion of the cochlear partition deflects hair bundles, opens mechanoelectrical transduction channels to permit K^+^ entry, and thus depolarises the hair cells [20, 37, 40]. Aside from triggering the release of a neurotransmitter by inner hair cells, the electrical excitation elicited by displacement of the hair bundles changes the length of cell bodies of outer hair cells. This phenomenon is called somatic motility or electromotility; it depends upon the motor protein prestin [14, 52] and can mechanically amplify cochlear partition oscillations [31, 33].

A weak pure tone leads to a vibration of the cochlear partition that is greatly amplified near the site of the maximal vibration. Louder tones are progressively less amplified, resulting in a nonlinear compression of the vibration magnitude with respect to sound intensity [21, 43]. The nonlinear compression can be measured through laser interferometry and permits the detection of a million-fold range of sound pressure in normal hearing [33, 45]. For guinea pigs, cats, and chinchillas stimulated at 40 to 90 dB SPL, the response is related to the level of stimulation through a power law with a logarithmic slope of approximately one-third [43]. This property is likely to mirror the observed third-order change in open probability of the mechanoelectrical transduction channels as a function of the hair bundle deflection in outer hair cells [30, 32]. Moreover, the nonlinear behaviour of the cochlear partition leads to nonlinear distortion and distortion-product otoacoustic emissions (DPOAEs), among which the cubic ones are dominant [40, 43].

Recently, however, Doppler vibrometry combined with optical coherence tomography (OCT) has demonstrated quadratic distortion in the vibration profile of the region including outer hair cells but not in the vibration profile of the basilar membrane near the cochlear base [35, 49]. A quadratic non-linearity can create a static offset in response to a pure tone. In support of this notion, the electrophysiological recordings from the basal turn have demonstrated an offset in the sinusoidally oscillating membrane potential of outer hair cells and in the cochlear microphonic potential, which primarily mirrors the current flowing through the mechanosensitive channels [12, 22]. In addition, in the apical turn, a static offset of 1–200 nm in the vibration of the cochlear partition has been measured with a laser interferometer [7]. Similar motion has also been detected from Reissner’s membrane and from the tectorial membrane overlaying hair cells [9, 42]. Nevertheless, none of the studies involving OCT vibrometry have yet described slow vibrations at frequencies below 100 Hz in the partition, possibly owing to a limitation of the methodology or of the algorithm of data analysis [11, 27, 28, 35, 49].

In this study, we therefore aimed to directly measure and investigate the static offset in the vibration of the cochlear partition in the basal turn of live guinea pigs. For this purpose, we modified sinusoidal phase modulating (SPM) laser interferometry, a technique that can accurately detect the target’s movement at low frequencies including 0 Hz at a high signal-to-noise ratio [46]. Our in vivo measurements confirmed the presence of a nanoscale offset in the travelling wave. Moreover, we found that the magnitude depended upon the intensity and frequency of the stimulation. Finally, pharmacological experiments implied the involvement of somatic motility by outer hair cells in the offset.

## Materials and methods

### The laser interferometer

The laser interferometry used in this study was developed on the basis of the SPM method [46]. The interferometer was integrated into a commercial microscopy system (BX61WI; Olympus, Tokyo, Japan) as shown in Supplementary Fig. 1a (Online Resource 1). A light source was provided by a near-infrared laser diode (wavelength 780 nm; linewidth 2 MHz; maximum power 15 mW; LP780-SAD15; Thorlabs, NJ, USA). This laser diode contained an optical isolator and optical fibre. The light intensity at the end of the fibre was fixed at 12.6 mW by a laser controller (CLD1011; Thorlabs, NJ, USA). A parallel beam of light was emitted from a collimator lens (F810APC-780; Thorlabs, NJ, USA) connected to the fibre. This arrangement reduced the light intensity to 11.1 mW. The parallel light was divided into two beams in beam splitter I (90/10 Beamsplitter; Chroma, VT, USA); one beam was provided for the interferometer (9.99 mW), and the other was directed towards a CMOS camera (AdvanCam-18HR; Olympus, Tokyo, Japan). The former was split by beam splitter II (CCM1-BS014/M; Thorlabs, NJ, USA) equally into a reference beam and object beam, which were used to irradiate a reference mirror (NE530B; Thorlabs, NJ, USA) and a sample object, respectively, through objective lenses (XLFluor 4×/340; Olympus, Tokyo, Japan). Each lens was placed at a distance of 2.8 cm from the mirror or the object. The light intensity measured at the termination of each beam was 3.3 mW. The beams that were reflected from the mirror and sample were coupled together in beam splitter II and were detected by interference at a photodetector (PDA100A-EC; Thorlabs, NJ, USA) that was placed at a distance of 24 cm from the beam splitter. The gain and the low-pass cut-off frequency of the detector were set to 40 dB (× 100) and 225 kHz, respectively. A piezo-actuator (AE0505D18; Thorlabs, NJ, USA) was attached to the reference mirror. In this context, the actuators that we used originated from two different lots, A and B. To sinusoidally move the mirror, the actuator was stimulated with the AC voltage evoked by means of a function generator (AFG-1022; Tektronix, OR, USA) as follows: for lot A, the frequency was 45 kHz and the peak-to-peak voltage was 11, 13, 15, 17, or 20 V (the experiments in Supplementary Figs. 1 and 2 and experiments on the four animals: GP#235, 257, 258, and 396); for lot B, the frequency was 49 kHz and the peak-to-peak voltage was 20 V (the other experiments). In any case, the initial phase was set to 60° or 70°. The induced vibration amplitude was 16.9–25.0 nm. The function generator was driven by a trigger pulse generated by a computer that had a sampling rate of 204.8 kHz (NI PXI-4461; National Instruments, TX, USA). The pulse generation was controlled by a software that we wrote in LabVIEW (LabVIEW™ 2013 Service Pack 1, 32-bit; National Instruments, TX, USA).

Note that in this study, we modified the SPM method to simultaneously measure the sinusoidal vibrations and offset in the target. The details of this arrangement are described in Online Resource 2.

### Performance evaluation experiments

The accuracy of measurement of the offset with the modified SPM method (see Online Resource 2) was verified with a piezo-actuator attached to a strain gauge (PZS001; Thorlabs, NJ, USA). This actuator expanded in parallel with the direction of the applied DC voltage. An AC voltage of 21 kHz, 0.28 V peak to peak, and a DC voltage of 0, 0.025, 0.05, 0.075, 0.1, or 0.25 V were simultaneously applied to the actuator for 18 ms as electrical stimulation (Supplementary Fig. 1b). The difference in the actuator’s length recorded for 5 ms before and during the stimulation was quantified by a strain gauge method. The resistance in the gauge was converted by an amplifier (AMP002; Thorlabs, NJ, USA) to voltage, which was analysed on a commercial strain gauge reader (KSG101; Thorlabs, NJ, USA). At each voltage, the actuator was stimulated 80 times, and the average of the measurements was taken as a data point. Subsequently, the change of the actuator’s length was examined by the modified SPM method too, with the same stimulus protocol as in the strain gauge method and the workflow described in Supplementary Fig. 1e and in Supplementary Text in Online Resource 3. Finally, the values obtained by the two methods were compared (Supplementary Fig. 1f and g).

Additional evaluation for the modified SPM interferometry was carried out as follows. Suppose that the sample is sinusoidally vibrated, and its amplitude is gradually enlarged without the offset. In homodyne vibrometry, when the sample’s vibration amplitude is close to *λ*/8, i.e. approximately 100 nm in our system (*λ* = 780 nm), a nonlinear relation is observed between the change of interference signal intensity and that of the vibration amplitude [25, 29]. This non-linearity may apparently induce an offset in the measurement. Nonetheless, we confirmed that this artefact was barely detectable by the SPM interferometry when the vibration amplitude of a piezoelectric element ranged from approximately 0.1 to 50 nm (Supplementary Fig. 2a). Indeed, in this experiment, the observed background offset values fell within a small range of ± 0.5 nm (Supplementary Fig. 2b). It is noteworthy that the measured vibration amplitudes of acoustically stimulated cochlear partitions never exceeded 50 nm.

### The animal experiments

In accordance with the ethics guidelines (see Compliance with ethical standards), guinea pigs (see the next subsection) were housed at the animal facility in the Niigata University School of Medicine. The animals were kept on a 12 h light/12 h dark cycle and were provided with ad libitum access to food and water. All the experiments were conducted during the light phase. The animal handling and reporting complied with the ARRIVE guidelines [26].

### Animal preparation for in vivo experiments

For in vivo assays, 141 male Hartley guinea pigs were used (200–580 g; 2–8 weeks old; SLC, Shizuoka, Japan). All the following experiments were carried out in an acoustically and electrically shielded room. Guinea pigs were deeply anesthetised with intra-peritoneal injection of urethane (1.5 g kg^−1^). The toe pinch, corneal reflexes, and respiratory rate were examined to evaluate the depth of anaesthesia. When anaesthesia was insufficient, additional urethane was injected into the animals at 0.3 g kg^−1^. During the experiment, the body temperature of each animal was kept at 38 °C by means of a heating pad (Deltaphase Isothermal Pad; Braintree Scientific, MA, USA). The temperature of the cochlea was additionally controlled by supplemental heat to the head from a lamp [34]. After tracheotomy, which was intended for the maintenance of spontaneous breathing, an anterior portion of the bulla was opened with a ventral approach to expose the basal turn of the cochlea [36]. Then, the tensor tympani muscle was cut with a sharp scalpel [33]. Additionally, a fenestra was made on the lateral site of the bulla in order to shine the laser light onto a spot of the basilar membrane constituting the surface of the cochlear partition through the round window membrane. The pinna was resected. The head of a guinea pig was fixed on an acrylic plate (50 × 20 × 5 mm) with two M2.5 screws. The plate was tightly connected to the stage (SL20/M; Thorlabs, NJ, USA). In this process, the angle and position of the cochlea were manually controlled to let the laser beam irradiate the cochlear partition through the round window membrane as perpendicularly as possible.

### Acoustic stimuli

To examine the auditory brainstem response (ABR) and motions in the cochlear partition, tone-burst sounds were generated from a speaker (EC1; Tucker-Davis Technologies, FL, USA) with its driver (ED1; Tucker-Davis Technologies, FL, USA). The driver was controlled by the application of AC voltage between 0.1 mV and 10 V. The input port of a Y-shaped waveguide was connected to the exit of the speaker. This set-up enabled us to symmetrically divide the propagation route of the sound into two paths. Each output port of the waveguide was attached to an ear tip (ER10D-T03; Etymotic Research Inc., IL, USA). One tip was tightly inserted into the left external ear canal of an animal. The other tip was next connected to a different type of ear tip (ER10D-T013; Etymotic Research Inc., IL, USA), which had been assembled with a microphone (ER-10B+; Etymotic Research Inc., IL, USA). This microphone, which had been calibrated beforehand with a sound calibrator (Type2127; ACO CO, LTD, Tokyo, Japan), was employed to measure the intensity of the sound from the speaker. Protocols of acoustic stimuli for each series of the assays are described in Supplementary Fig. 3a and b, Supplementary Text in Online Resource 3, and below.

### Measurement of motions in the cochlear partition

Before the tensor tympani muscle of each guinea pig was cut and after measurement of the motions in an intact cochlear partition, the ABR was examined as previously described [47] (see Supplementary Table 1). The procedure for ABR measurement is described in Supplementary Methods (Online Resource 2).

The motions of the cochlear partition were analysed by the modified SPM interferometry as follows. A laser beam was directed through the round window membrane to the cochlear partition 1.5–2.0 mm apically from the basal extreme of each cochlea [29] (Fig. 1a). The target for the beam was determined by the appearance of a shadowed belt, which is likely to correspond to the region of outer hair cells [4] (Fig. 1b). The illuminated area was approximately 900 μm^2^. Although the laser was focused upon the basilar membrane, interference signals were theoretically evoked from not only the membrane but also the other components of the cochlear partition; the signals were gathered in the photodetector of the modified SPM interferometer. The protocols and workflow for measurement of the motions in the cochlear partition are illustrated in Supplementary Figs. 1e, 3a, and b and in the Supplementary Text in Online Resource 3. Of note, before examination of the partition’s motion in each cochlea, we focused the microscope on the round window membrane and confirmed that the voltage of the interference signal detected at the same frequency as that of acoustic stimulation did not exceed the limit of detection (LOD) [29].

**Fig. 2:**
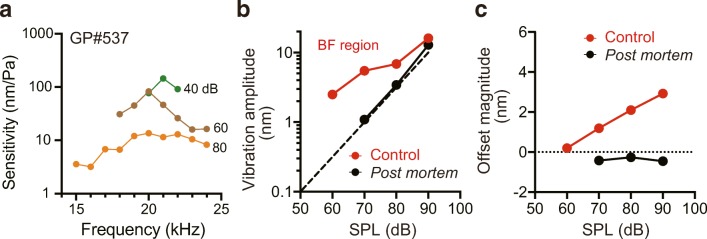
Measurement of an offset in the cochlear partition. For this guinea pig (GP#537), the tuning curves were initially obtained with pure tone-burst sounds at 40, 60, and 80 dB SPL (**a**). At each intensity, the frequency was changed from 15 to 24 kHz in 1 kHz steps. The stimulus protocol was the same as that illustrated in Fig. 1c and d (see Supplementary Fig. 3a). In accordance with the threshold tuning curve, the best frequency (BF) for this animal was found to be 21 kHz. The vibration amplitude and offset magnitude were then simultaneously recorded in the same animal under control (red) and post mortem (black) conditions; the respective results were plotted in panels **b** and **c** as a function of the sound pressure level (SPL). In panel **b**, although the amplitude of sinusoidal vibrations grows linearly post mortem, under control circumstances, the data fit a power law relation with a slope of approximately 0.3 dB/dB indicative of compressive non-linearity. The dashed line in **b** indicates linear growth. Note that in panel **c**, the offset magnitude under control conditions monotonically increases as the stimulus increases; this behaviour clearly differs from the post mortem response that represents the noise level of the measurements. The data depicted in panels **b** and **c** were acquired according to the workflow described in Supplementary Fig. 1e; a stimulus cycle consisted of a tone-burst sound with a 1-ms rising phase, 16-ms duration, and a 1-ms falling phase, followed by an 82-ms interval (see Supplementary Fig. 3b). The summary flowchart for this series of experiments is displayed in Supplementary Fig. 3c

**Fig. 3:**
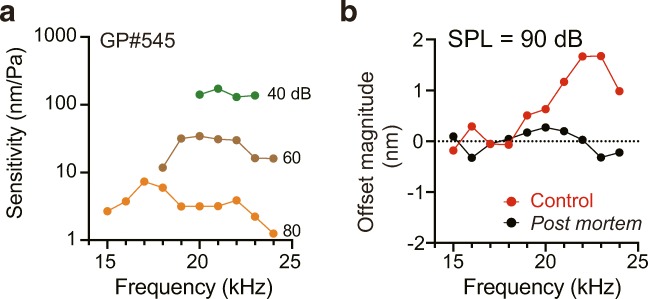
Frequency dependence of the offset. In this series of experiments, a guinea pig (GP#545), whose BF was found to be 21 kHz by means of the tuning curves obtained in advance (**a**), was exposed to 90 dB acoustic stimuli at different frequencies (15–24 kHz in 1 kHz increments), and motions of the cochlear partition were measured by the SPM interferometry. In panel **b**, the offset magnitudes are plotted as a function of stimulus frequencies. Under control conditions, the offset was prominent at the stimulus frequencies that elicited strong non-linearity in panel **a** (21–24 kHz); at other frequencies, the responses diminished to the level similar to that of the post mortem measurements, which represents the noise floor. For the tuning curves (**a**), acoustic stimuli of 40, 60, or 80 dB SPL at varying frequencies (15–24 kHz in 1 kHz steps) were tested with the protocol illustrated in Supplementary Fig. 3a. The data depicted in panel **b** were acquired according to the workflow described in Supplementary Fig. 1e; a stimulus cycle consisted of a tone-burst sound with a 1-ms rising phase, 16-ms duration, and a 1-ms falling phase, followed by an 82-ms interval (see Supplementary Fig. 3b)

As presented in Fig. 1c and d, a tuning curve was constructed for a guinea pig to verify our animal preparation and experimental approach (for details, see Supplementary Text in Online Resource 3). In other in vivo experiments, the offset of the cochlear partition was characterised through the following process. Firstly, for an individual guinea pig, tuning curves were constructed in advance by the stimulus protocol illustrated in Supplementary Fig. 3a (40, 60, and 80 dB SPL; 10 s in total for stimulation) to identify the best frequency (BF) [18, 39] (for the definition of the BF, see the ‘Results’ section). Secondly, the LOD of the interference signals at the BF was evaluated by means of 80 sets of 5 ms recordings without stimulation. The third step was the measurement of the offset. The acoustic stimulation consisted of a tone-burst sound with a 1-ms rising phase, 16-ms duration, and a 1-ms falling phase, followed by an 82-ms interval; the 5-ms data recorded in the absence and presence of the stimulation were analysed to characterise the motions in the cochlear partition (Supplementary Fig. 3b). For the stimulation at the BF, the intensity was varied from 50 to 90 dB SPL in 10 dB increments (Fig. 2b and c). Alternatively, for examination of frequency dependence of the offset (Fig. 3b and Supplementary Fig. 4), the sounds at a constant intensity of 90 dB SPL were applied to an animal while the frequency was changed (15–24 kHz; 1 kHz steps). All these offset measurements were carried out by means of basically the same procedure and workflow as those used for the pilot experiment in Supplementary Fig. 1c–e.

**Fig. 4:**
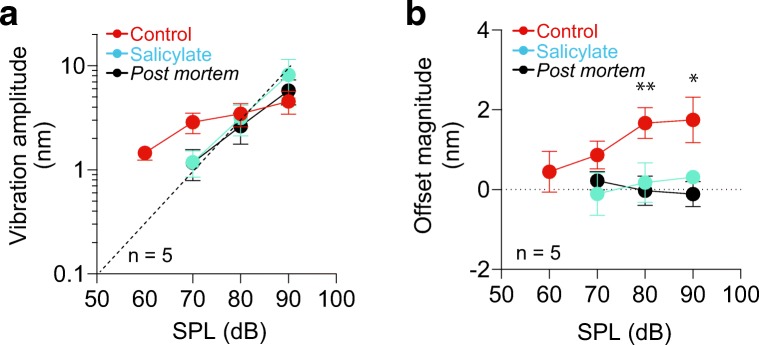
Effects of pharmacological perturbation of somatic motility in outer hair cells. The sound-evoked motions of the cochlear partitions were measured by means of the SPM interferometer in five guinea pigs under control (red), salicylate administration (blue), and post mortem (black) conditions as indicated in the insets. The stimulus frequency was constant at 21 kHz. The procedure for the pharmacological perturbation is described in the main text. In panels **a** and **b**, dots and error bars (mean ± SD) denote the amplitude of sinusoidal vibrations (**a**) and the magnitude of the offset (**b**), which are plotted as a function of sound pressure levels (SPLs; *n* = 5). In panel **a**, the vibration amplitude obtained under control conditions is characterised by compressive non-linearity; therefore, 21 kHz is likely to preserve the effects of the BF in these animal preparations (see Fig. 2b). The amplitude measured post mortem and during salicylate treatment shows a passive response and loses the property of compressive non-linearity. The dashed line in panel **a** indicates linear growth. In panel **b**, the individual values of the offset detected with stimuli of different intensities under the three conditions were plotted and statistically compared by the paired *t* test with Bonferroni’s correction for multiple comparisons (*p < 0.05, **p < 0.01; control versus salicylate treatment *p* = 0.1659, *t* = 2.679 for 70 dB, *p* = 0.0054, *t* = 7.396 for 80 dB, *p* = 0.0312, *t* = 4.555 for 90 dB; salicylate treatment versus post mortem conditions *p* = 0.8637, *t* = 1.225 for 70 dB, *p* = 0.8094, *t* = 1.280 for 80 dB, *p* = 0.0999, *t* = 3.188 for 90 dB). This series of data (**b**) points to a prominent reduction in the offsets by euthanasia or by the application of salicylate. All the individual measurements used for the analyses of **a** and **b** are displayed in Supplementary Fig. 5

Out of the 141 guinea pigs examined in this study, we excluded animal preparations and a series of acquired data from the analyses under any one of the following nine conditions. Firstly, the experiment was terminated when the ABR threshold measured before cutting of the tensor tympani muscle exceeded 30 dB SPL (25 animals) (for details, see Online Resource 2). The second condition was the case where we found a rupture in the ear drum when dissecting the tensor tympani muscle (four animals). The third issue was low intensity of interference signals from the cochlear partition at the outset of each experiment; this problem was attributed to obstruction of laser irradiation of the target position by an overlay of a styloid process upon the round window membrane (27 animals). Fourthly, we discarded the preparations in which stimulus intensity for acquisition of data for the threshold tuning curve was ≥ 60 dB SPL (16 animals). In the fifth condition, during the measurements of the cochlear partition, the object’s interference signals (Supplementary Fig. 1d) induced by 60 dB stimuli did not exceed the LOD under control conditions (three animals). In the sixth case, the ABR threshold measured immediately before euthanasia of an animal (Figs. 2 and 3) or administration of salicylate crystals (sodium salicylate; Wako, Osaka, Japan; Fig. 4) exceeded 50 dB SPL (six animals). Seventhly, total experiment time for an animal exceeded 4 h (one animal), the time limit for keeping a guinea pig’s conditions stable [19]. Eighthly, in the tuning curves, the peak position for the amplitude of sinusoidal vibrations did not shift to a lower frequency as the stimuli were intensified (seven animals). Finally, 42 animals died during the experiments. Accordingly, the number of the animals described in the ‘Results’ section of the main text is 10.

### Statistical analysis

Means ± SD served as descriptive statistics. Statistical significance of the data in Fig. 4 was determined with the paired *t* test (Prism 8; GraphPad Software, San Diego, CA, USA). Data with a *p* value of < 0.05 were considered significant. The *p* values were subjected to Bonferroni’s correction for multiple comparisons.

## Results

### Detection and characterisation of the offset in the cochlear partition

We constructed an interferometer by combining two previously described methodologies: the SPM technique, which is intended for the measurement of static offsets [46], and homodyne interferometry, which quantifies sinusoidal vibrations [25] (Supplementary Fig. 1a–e). Although few applications of the original SPM method have been reported, experiments with a piezoelectric element confirmed the accuracy and efficacy of our interferometer for quantitative detection of offsets (Supplementary Fig. 1f and g). Interferometry could determine in which direction a sample’s position moved: offsets towards the scala vestibuli or the scala tympani were represented by positive or negative values, respectively (see Online Resource 2).

By means of the interferometer we constructed, we sought to examine sound-induced motions in cochlear partitions in the high-frequency region of guinea pig cochleae. The amplitude of the partition’s sinusoidal vibrations at the basal turn of the cochlea is much less than that at the apical turn [43]. Because we expected that the offset of the target region would be relatively small and affected by even subtle perturbations, we adopted a minimally invasive experimental approach. A laser beam was directed through the transparent round window membrane at the cochlear partition 1.5–2.0 mm apically from the basal extreme of an intact cochlea [29] (Fig. 1a and b). In addition, we established criteria for successful measurements as described in the ‘Materials and methods’ section. The properties of the tuning curve obtained for sinusoidal vibrations with this experimental approach and tone-burst stimuli composed of pure sounds (10–30 kHz) are similar to those described elsewhere [10, 51] (Fig. 1c and d).

Then, in a different guinea pig, we intended to stimulate the intact cochlea with pure tone-burst sounds at the BF, which induced a maximal amplitude in the threshold tuning curve constructed in advance [18]. Recording of the tuning curves by the same stimulus protocol as that depicted in Fig. 1 (10–30 kHz, 10 dB increments; Supplementary Fig. 3a) took at least 2–3 h; therefore, a series of experiments including animal preparation with the surgical procedure and measurement of the partition’s motions under control conditions could not be completed within 4 h, the time limit that ensured an animal’s intact hearing threshold [19]. We therefore modified the protocol for tuning curves as follows. Firstly, the range of the stimulus frequencies was narrowed to a window of 15–24 kHz. Secondly, the sound intensity was increased in 20 dB steps from 40 dB SPL. The BF determined by this procedure was 21 kHz under control conditions (Fig. 2a). Next, in the same animal, we succeeded in measuring not only sinusoidal vibrations but also the offset with a different stimulus protocol (Fig. 2b and c; for the protocol, see Supplementary Fig. 3b). The threshold intensity for detection of sinusoidal vibrations was 60 dB SPL, which exceeded the level in the evaluation experiment (40 dB SPL; see Figs. 1c, d and 2a). This impaired sensitivity was attributed to an elevation of the detection limit by the ultrashort recording period (5 ms; Supplementary Fig. 3b), which minimised the influence of low-frequency noise such as breathing and heartbeats in live animals. The vibration amplitude grew less steeply as sound pressure increased from 60 to 90 dB, manifesting the typical profile of compressive non-linearity (Fig. 2b) [43]. During stimulation at 60 dB SPL, we simultaneously detected an offset of 0.2 nm towards the scala vestibuli (Fig. 2c). As the sound intensified, the magnitude was likely to increase monotonically (1.2 nm at 70 dB SPL, 2.1 nm at 80 dB SPL, and 2.9 nm at 90 dB SPL).

After the animal had been euthanised, the amplitude of the sinusoidal vibrations evoked by stimulation (21 kHz) grew linearly with sound pressure (Fig. 2b) [5, 6]. In addition, the offset decreased markedly for stimuli of 70–90 dB SPL; the magnitude was − 0.4 nm at 70 dB, − 0.3 nm at 80 dB, and − 0.5 nm at 90 dB (Fig. 2c). In this situation, 60 dB stimuli did not induce a motion exceeding the threshold of detection. These observations indicate that the offset is dependent upon cellular activity sensitive to a loss of metabolic energy.

We further examined the effects of changing stimulus frequency upon the offset. A guinea pig, whose BF was found in advance to be 21 kHz (Fig. 3a), was exposed to 90 dB pure-tone sounds at different frequencies (15–24 kHz; for the stimulus protocol, see Supplementary Fig. 3b). The offset peaked at 23 kHz and decayed on both sides of the peak (Fig. 3b). Clear responses that exceeded 1.0 nm were observed at ≥ 21 kHz (Fig. 3b). In the sinusoidal vibrations within this range, we observed prominent non-linearity as well as sensitivity exceeding 100 nm Pa^−1^ at 40 dB SPL (see Fig. 3a). On the other hand, the offsets detected at ≤ 18 kHz were subtle: − 0.2 to 0.3 nm. These values approximately fell into the range that was determined post mortem in the same animal (from − 0.3 to 0.3 nm). Similar results were observed in two other animals (Supplementary Fig. 4). Accordingly, it seems probable that the offset is evoked at frequencies near the BF, and the magnitude depends upon stimulation frequency.

### The mechanism underlying the offset of the cochlear partition

We attempted to identify the factor that critically contributes to the offset detected in the motion of the cochlear partition. Previous measurements by OCT vibrometry indicated that the quadratic distortion product in response to tone-complex stimuli at multiple frequencies occurs in a region including outer hair cells [35, 49]. In addition to this observation, the disappearance of the offset post mortem (Figs. 2c and 3b) prompted us to examine the involvement of the active process of outer hair cells, which is responsible for the compressive non-linearity observed in sinusoidal vibrations of a healthy basilar membrane [33]. Because somatic motility in hair cells plays a key role in the active process and depends on the motor protein prestin [52], we pharmacologically blocked this protein’s activity and compared the partition’s motions with those under control and post mortem conditions (Fig. 4).

In this series of assays as well, it was necessary to complete all the recordings in individual live animals within 4 h [19]. To shorten the time of the experiment, we therefore omitted the determination of tuning curves and used 21 kHz pure tone-burst sounds for stimulation. This frequency was expected to be close to the BF in the target region for the laser beam, according to the tuning curves in the foregoing four guinea pigs (Figs. 2 and 3 and Supplementary Fig. 4). Under control conditions, we again clearly observed not only the compressive non-linearity of the vibration amplitude, a typical phenomenon detected with stimuli at the BF, but also a significant offset in five animals (Fig. 4a and b; Supplementary Fig. 5). Note that the offset magnitude increased monotonically with sound pressure; this trend was similar to that obtained in Fig. 2c with stimuli at the measured BF (21 kHz). Crystals of salicylate, a blocker of prestin, were subsequently placed onto the round window membranes in the individual cochleae [31, 41]. Twenty minutes later, the motions of the partitions were again examined. The amplitude of the sinusoidal vibrations depended linearly upon the strength of stimulation [31] (*n* = 5; Fig. 4a). In addition, the offset towards the scala vestibuli was prominently attenuated at 80 and 90 dB SPL (Fig. 4b; control versus salicylate administration for the offset; *p* = 0.0054 and *t* = 7.396 at 80 dB, *p* = 0.0312 and *t* = 4.555 at 90 dB; according to the paired *t* test with Bonferroni’s correction for multiple comparisons). The values of the two parameters at 70–90 dB SPL with the pharmacological perturbation were comparable with the level characteristic of post mortem conditions (Fig. 4a and b). The offset is therefore likely to depend on the somatic motility of outer hair cells.

## Discussion

Cooper and Rhode [8] placed reflective microbeads on the basilar membrane at the basal turn of the guinea pig cochlea and examined the travelling wave by means of an interferometer. When stimulating this cochlear portion with pure-tone sound at a high frequency, they detected a negligible static offset, which can be defined as 0 Hz motion. Recent studies using OCT systems indicate that at the basal turn, the quadratic distortion is produced by stimuli in the region comprising outer hair cells [35, 49], implying the presence of the offset in these cells. As for multi-tone sounds used in such works, different frequency components should induce individual offsets, and these responses must be mixed. To quantitatively assess the phenomenon of a static offset, we chose in this study stimulation at a single frequency. The SPM interferometry system that we utilised has a prominent property: the algorithm designed for the measurements and data analyses allowed us to extract slow motions of 0–100 Hz, which have not yet been addressed by OCT vibrometers. Our system automatically discarded significant background movements induced by heartbeats, respiration, and muscle contractions from the measurements in real time to improve the signal-to-noise ratio of the data (see Supplementary Methods in Online Resource 2). Due to this advantage, our interferometer was suitable for the purpose of this study, although the methodology used here could not determine which cell type(s) within the cochlear partition were involved.

Although the offset that we detected in the cochlear basal turn was only a few nanometres, it is likely to be significant for several reasons. In each animal, we could not determine the range of background fluctuations of the intact cochlear partition in a resting state because the recording period of the experimental protocol including this evaluation inevitably exceeded 4 h, which is the limit that guarantees an intact hearing threshold of these animals [19]. Nevertheless, in the 18 measurements under control conditions with stimuli of 70–90 dB SPL in the six guinea pigs analysed in Figs. 2 and 4, 15 data points for the offset exceeded 1 nm. In all 15 cases, the response was clearly impaired by salicylate treatment of the cochleae or by euthanasia; the magnitudes then fell into a range of − 0.8 to + 0.9 nm. Values of approximately ± 1 nm were likely to represent the maximal background noise and thus the threshold for accurate detection in our in vivo preparations. Accordingly, in the experiment presented in Fig. 3b, the offset measurements at ≥ 21 kHz—the range that included the BF—was significant. Moreover, during the pharmacological treatment and post mortem (Figs. 2c and 4b and Supplementary Fig. 5), the data at 60 dB SPL could not be plotted. The motion was likely to have been suppressed by each of the two perturbations to a level too low to be detected.

In the cochlear basal turn, the amplitude of the quadratic distortion product with stimuli of 94 dB SPL exceeded that for stimuli of 74 dB SPL [35]. In support of this finding, we observed that the partition’s offset grew monotonically with sound pressure (Figs. 2c and 4b; Supplementary Fig. 5). Moreover, the response peaked at a stimulus frequency near the BF (Fig. 3b and Supplementary Fig. 4). Similar intensity and frequency dependences of the offset are also observed for Hensen’s cells in the cochlear apical turn of guinea pigs [7]. Therefore, this pattern may be conserved throughout the cochlea. The maximal offset that we measured in the high-pitch region with stimuli of 90 dB SPL was approximately 3 nm for the BF of 21 kHz (Fig. 2c), which is much smaller than the value of 130 nm in the low-pitch region for BFs of 0.6–0.9 kHz [7]. This difference could be due to a gradient of the morphological and physical properties of the basilar membrane and the overlying cellular components along the cochlea [13]. Note that at the target portion of the apical turn, the offset is peaked at the stimuli of ~ 0.2 kHz, which is separated from the BF by 70–80% of this frequency [7]. On the other hand, we found that the gap was ~ 10% in the basal turn (Fig. 3b and Supplementary Fig. 4). The elements underlying this discrepancy remain uncertain and thereby would be analysed by further work.

Although the quadratic distortion product in the partition’s vibrations induced by multi-tone stimuli is likely to emerge from the activity of outer hair cells [35, 49], the underlying mechanism has not yet been identified. We therefore examined the effect on the partition’s motion of salicylate, which is commonly used as an inhibitor of somatic motility of outer hair cells [1, 22, 23, 38, 48]. The results with this reagent (Fig. 4) indicate that in response to acoustic stimulation, a steady contraction mediated by prestin occurs in the hair cell soma, and this action likely underlies the offset of the cochlear partition. Nevertheless, the pharmacological targets should be addressed carefully because of the following observations. In outer hair cells, somatic motility depends upon membrane potential [3, 23]. This electrical property is controlled by cation influx through mechanosensitive channels localised at the tip of the hair bundle [24]. Because application of salicylate reduces the amplitude of the channels’ current to some extent in vitro [24], this compound might affect active hair bundle motility. Moreover, administration of the compound attenuates blood flow in the vessels serving the lateral cochlear wall, which is connected with the cochlear partition [16]. This effect might change physical properties of the lateral wall and thus contribute to the elimination of the offset by the drug. Further experiments are needed to clarify how much factor(s) other than the somatic motility of hair cells are involved in offset induction.

The measurements in Fig. 4 implied that a steady contraction of the outer hair cells occurs in vivo and contributes to the offset of the cochlear partition. How this cellular motion is induced remains elusive. One possibility is that acoustic stimuli yield an offset of hair bundles. Alternatively, somatic motility might be regulated by organisation of the activity of efferent neurons innervating the outer hair cells. In any case, the steady change of hair cells’ length should control cellular mechanical activity, and this modification should be fed back to the offset of the cochlear partition.

An observation in a classical electrophysiological experiment suggests a possible physiological role for the offset. The amplitude of the cochlear microphonic, which primarily represents the current flowing through outer hair cells [17], temporarily diminishes when positive hydrostatic pressure is applied to the perilymph in the scala tympani, a perturbation that likely shifts the cochlear partition towards the scala vestibuli [53]. The hair cell’s current excites the cells and thereby elicits somatic motility [2]. Our interferometric measurement showed that the partition’s offset towards the scala vestibuli was well detectable with stimuli of 80 and 90 dB SPL (Figs. 2c and 4b; Supplementary Fig. 5). The offset might accordingly affect the hair cell’s somatic motility and attenuate the amplification of the sinusoidal vibrations of the cochlear partition when an animal is exposed to loud sounds. This idea points to a contribution of the offset to enhancement of the nonlinear compression of the vibration amplitude and thus to the broad dynamic range associated with normal hearing. The second possible significance of the offset is its relation to the machinery that prevents damage to fine cochlear structures by loud sounds. A similar role is proposed for the acoustic reflex of the stapedial muscle in the middle ear, but this action is negligible at stimulus frequencies exceeding 5 kHz [44]. The offset in the cochlear partition could serve as a fundamental system of protection from acoustic trauma. These kinds of possible functional significance of the partition’s offset should be verified in future studies.

## Electronic supplementary material

ESM 1 (PDF 790 kb).

ESM 2 (PDF 594 kb).

ESM 3 (PDF 448 kb).
