# Supplementary material for: Characterisation of the static offset in the travelling wave in the cochlear basal turn
Source: Pflugers Arch. 2020 Apr 22;472(5):625–35. doi: 10.1007/s00424-020-02373-6 (PMC7239825; doi:10.1007/s00424-020-02373-6)
Supplement: Supplementary file 1 — (PDF 790 kb). [file 424_2020_2373_MOESM1_ESM.pdf]

Characterisation of the static offset in the travelling wave in the cochlear basal turn

Pflügers Archiv - European Journal of Physiology

Takeru Ota[1,†], Fumiaki Nin[1,†,*], Samuel Choi[2,3], Shogo Muramatsu[3], Seishiro Sawamura[1], Genki Ogata[1], Mitsuo P. Sato[4], Katsumi Doi[4], Kentaro Doi[5], Tetsuro Tsuji[5,#], Satoyuki Kawano[2,5], Tobias Reichenbach[6], Hiroshi Hibino[1,2,*]

[1]Department of Molecular Physiology, Niigata University School of Medicine, Niigata 951-8510, Japan.

[2]AMED-CREST, AMED, Niigata 951-8510, Japan.

[3]Department of Electrical and Electronics Engineering, Niigata University, Niigata 950-2181, Japan.

[4]Department of Otolaryngology, Kindai University Faculty of Medicine, Osaka 589-8511, Japan.

[5]Department of Mechanical Science and Bioengineering, Graduate School of Engineering Science, Osaka University, Osaka 560-8531, Japan.

[6]Department of Bioengineering, Imperial College London, London SW7 2AZ, United Kingdom.

[†]These authors contributed equally to this work.

[#]Present address: Department of Advanced Mathematical Sciences, Graduate School of Informatics, Kyoto University, Kyoto 606-8501, Japan.

***Correspondence and material requests**: *H. Hibino*, Department of Molecular Physiology, Niigata University School of Medicine, 1-757 Asahimachi-dori, Chuo-ku, Niigata 951-8510, Japan. Email: hibinoh@med.niigata-u.ac.jp, Tel.: +81 25-227-2071, Fax: +81 25-227-0460; *F. Nin*, Department of Molecular Physiology, Niigata University School of Medicine, 1-757 Asahimachi-dori, Chuo-ku, Niigata 951-8510, Japan. Email: nin@med.niigata-u.ac.jp, Tel.: +81 25-227-2073, Fax: +81 25-227-0460.

Electronic Supplementary Material

Online Resource 1: Supplementary Figures and Legends

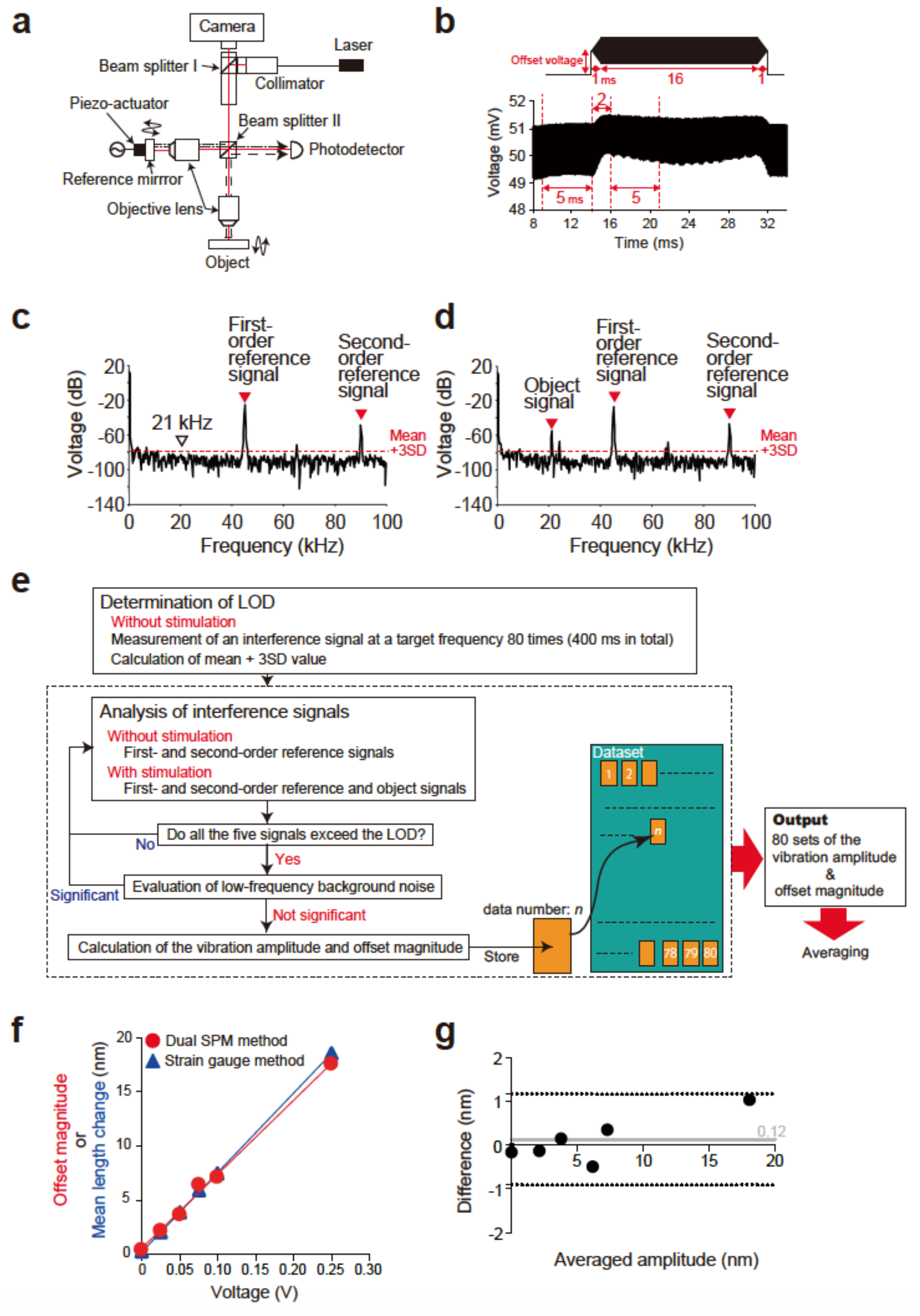

Supplementary Fig. 1 Modified sinusoidal phase modulating (SPM) interferometry. (a) A schematic of the modified SPM interferometer. (**b**) Time domain signals acquired in the photodetector (*lower panel*). A piezoelectric test element was electrically stimulated via the protocol described in the *upper panel*. A stimulus (18 ms) consisted of an AC voltage of 21 kHz, 0.28 V peak to peak, with a rising phase of 1 ms and

a falling phase of 1 ms as well as a DC offset voltage of 0.2 V, followed by an 82 ms interval. The 5 ms signals immediately before the onset of the stimulus and those at the time points 2–7 ms after the onset were analysed to determine the length change of the element (*lower panel*). (**c** and **d**) Raw data on the frequency domain. In a resting state of the piezoelectric element, two different signals that originated from the vibration property of the reference mirror (45 kHz) were observed at 45 and 90 kHz (**c**: first- and second-order reference signals). Upon stimulation (*upper panel* in **b**), an additional signal (the object signal in **d**) appeared at 21 kHz. *Red dashed* lines in both panels denote the means + 3 SDs, which represent limits of detection (LODs). A signal of 1 V was defined as 0 dB. (**e**) The workflow of data collection. The panels in the *dotted box* indicate the procedure and criteria of data acquisition in each cycle of stimulation. See **Supplementary Text** for details. (**f** and **g**) Comparison of the element's offset determined by the modified SPM method (*red*) with the mean length change measured by the strain gauge method (*blue*). The test target was stimulated with an AC voltage of 21 kHz, 0.28 V peak-to-peak, as well as various DC voltage levels, 0, 0.025, 0.05, 0.075, 0.1, or 0.25 V (*horizontal axis*) as shown in the *upper panel* of **b**. In **f**, the induced deformation was plotted as a function of DC voltage. Each data point is the average of the measurements in 80-cycle stimuli (for the data acquisition protocol, see **e**). The *solid* lines represent linear regression fitted to the data (slope = 68.456 nm/V, r^2 = 0.9961 for the SPM method; slope = 73.477 nm/V, r^2 = 0.9998 for the strain gauge method). *Panel* **g** illustrates Bland–Altman analysis of the difference between the SPM and strain gauge measurements; the differences of the former from the latter are plotted as a function of the mean of the two measurements. The *solid grey* line denotes the bias, namely, the mean difference (0.12), whereas the *dotted* lines indicate the 95% confidence interval, i.e. the range of 0.53 SD: –0.92 to 1.16 nm

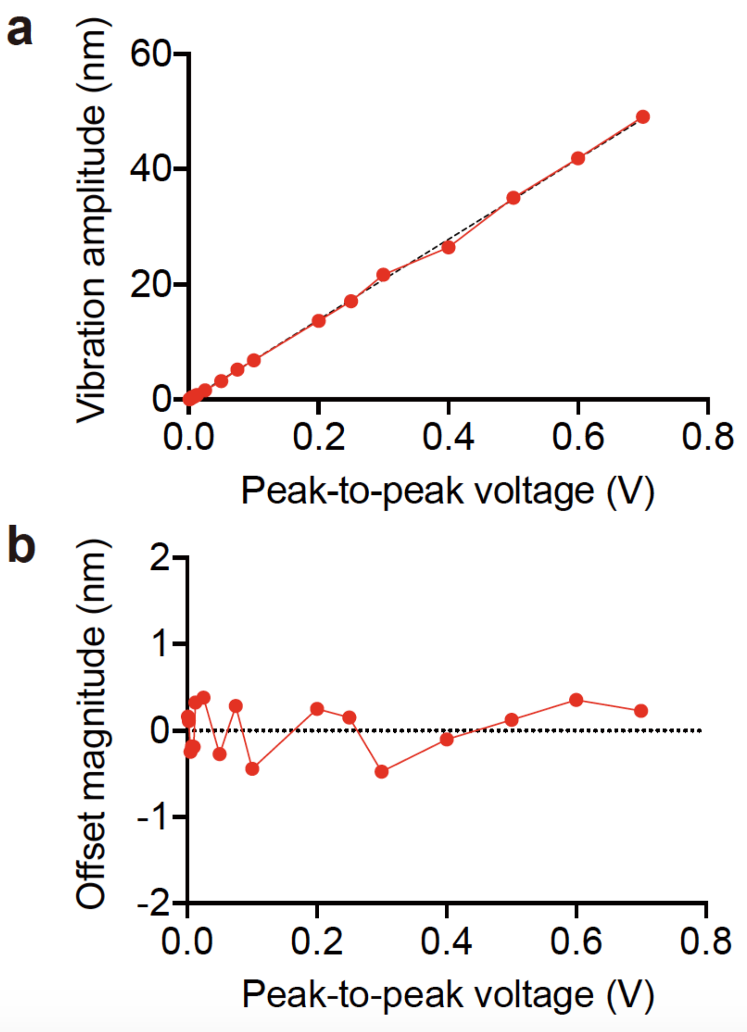

Supplementary Fig. 2 Evaluation of the modified SPM interferometry. In this experiment, a piezoelectric element served as a sample for the modified SPM interferometry and was electrically stimulated with the protocol described in the *top panel* of **Supplementary Fig. 1b**. Note that the DC offset voltage was always zero. The AC stimulus voltage (1 kHz) was 0.001, 0.0025, 0.005, 0.0075, 0.01, 0.0125, 0.025, 0.05, 0.075, 0.1, 0.2, 0.25, 0.3, 0.4, 0.5, 0.6, or 0.7 V (peak-to-peak). For data collection and analysis, the same workflow as that described in **Supplementary Fig. 1e** was used. Panel **a** shows the measured amplitude of the sample's sinusoidal vibrations as a function of the voltage applied. The *dotted line* indicates linear regression fitted to the data (r^2 = 0.9997). The magnitude of the simultaneously detected offset is plotted in panel **b.**

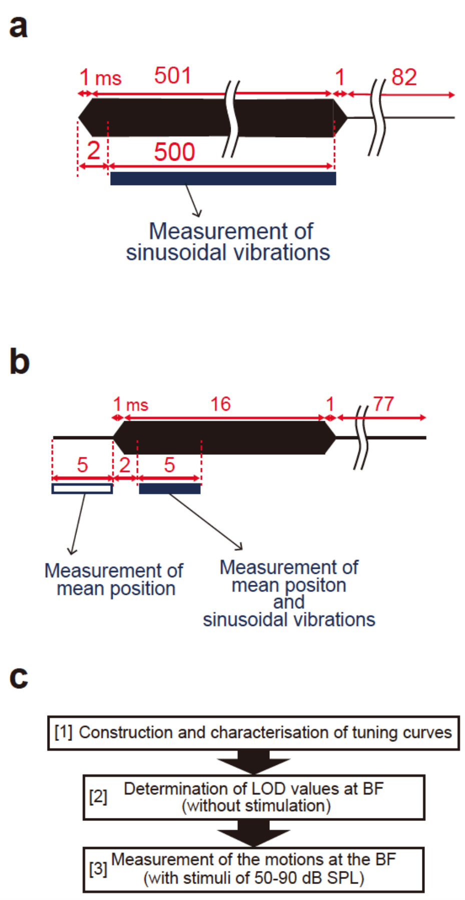

Supplementary Fig. 3 Acoustic stimulation and the experimental flowchart. (a) The stimulus protocol for the construction of tuning curves. A cycle consisted of a tone-burst sound with a 1 ms rising phase, 501 ms duration, and a 1 ms falling phase, followed by an 82 ms interval. The stimulation was applied 20 times to an animal. The interference signals recorded in the period indicated by a *bar* were used for measurements of the vibration amplitude. This protocol was used in the experiments presented in **Figs. 1c** and **d**, **2a**, and **3a** and in *left* panels of **Supplementary Fig. 4**. (**b**) The protocol for measurements of offsets. A stimulus cycle consisted of a tone-burst sound with a 1 ms rising phase, 16 ms duration, and a 1 ms falling phase, followed by an 82 ms interval. This stimulation was repeatedly applied to every animal in accordance with the workflow illustrated in **Supplementary Fig. 1e**. The periods for measurements of the vibration amplitude and offset are highlighted by *bars*. This protocol was employed for the data in **Figs. 2b** and **c**, **3b**, and **4** and in *right* panels of **Supplementary Fig. 4**. (**c**) The flowchart of data collection for offset measurements. Firstly, the data for tuning curves were acquired in individual guinea pigs by the stimulus protocol displayed in panel **a** (box [1]). As described in the *main text* and in the captions of **Figs. 2a** and **3a**, the intensities and frequencies of the stimulation were modified in each series of the assays. The results were subjected to determination of the best frequency (BF). Next, the animals were stimulated with BF or near-BF sounds of different intensities to examine the motions as presented in the panel (boxes [2] and [3]). In these measurements, a stimulus protocol described in **b** and the procedure shown in **Supplementary Fig. 1e** were used

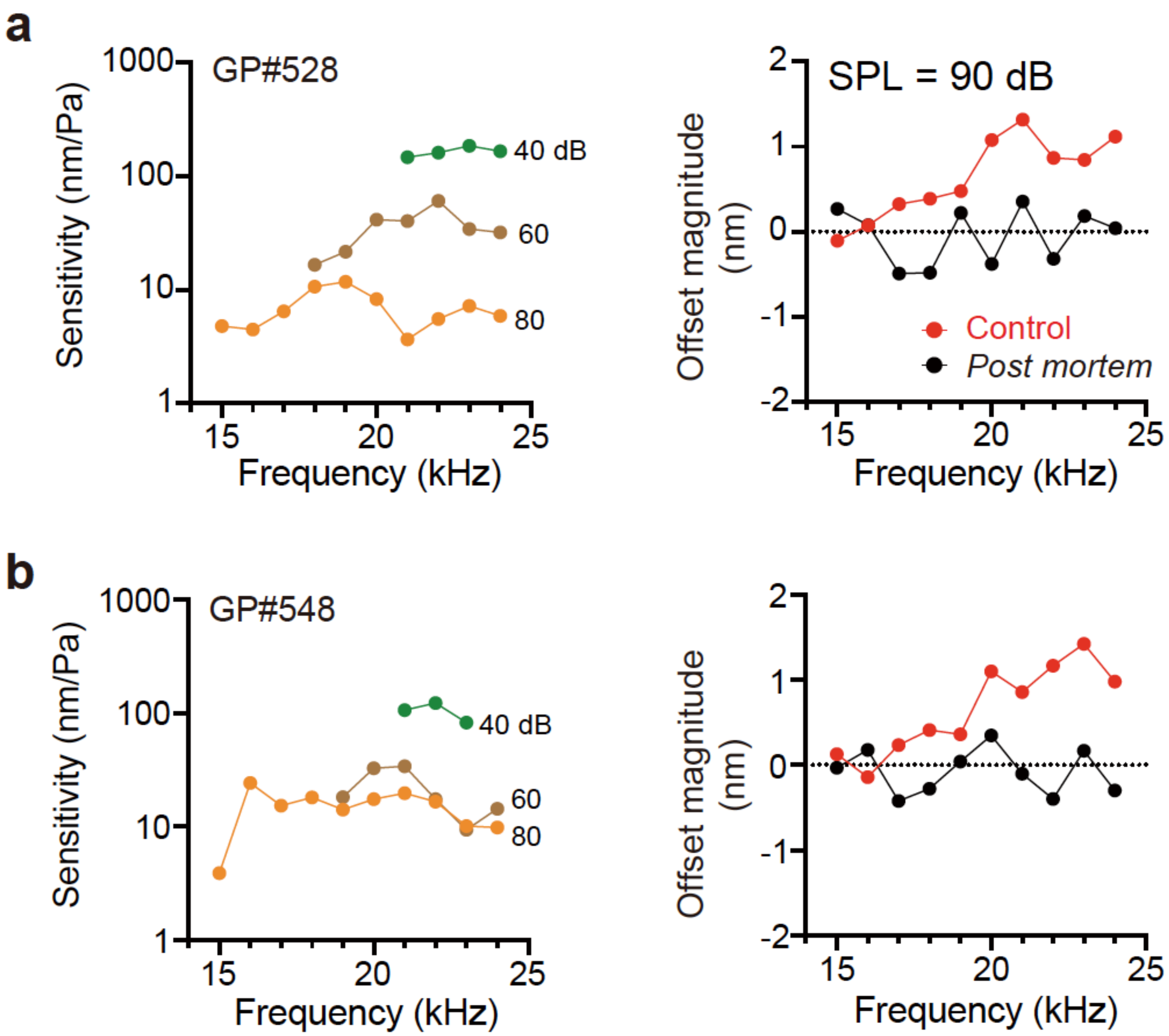

Supplementary Fig. 4 Frequency dependence of the offsets in other animals. Two individual guinea pigs (GP#528 in panel **a** and GP#548 in **b**) were examined by means of the SPM interferometer to characterise the dependence of the offsets on frequency. The experimental procedure and stimulus protocol in these experiments were the same as the one in the assay depicted in **Fig. 3**. Note that the stimulus intensity was a constant 90 dB sound pressure level (SPL). Tuning curves illustrated in *left* panels were employed to determine the BF: 23 kHz in case **a** and 22 kHz in case **b**. In the *right* panels, the offset magnitudes measured under control and *post mortem* conditions are plotted as a function of stimulus frequency

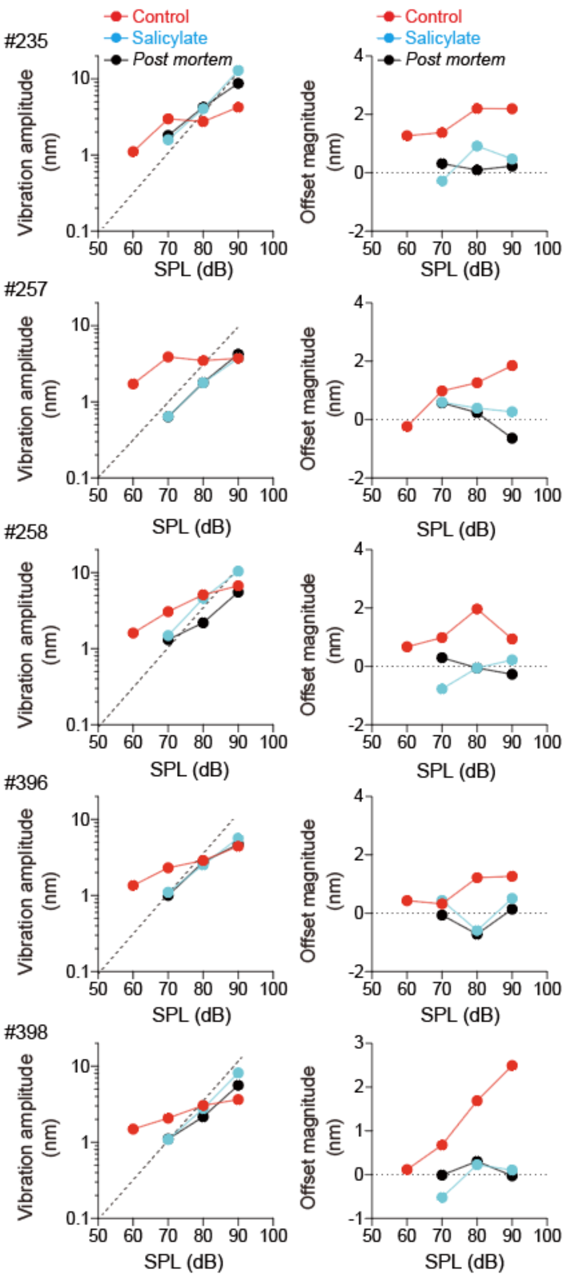

Supplementary Fig. 5 Individual data acquired in the test of the effects of salicylate. In each series of experiments, the sinusoidal vibrations and offsets in the guinea pig cochlear partition were simultaneously detected by means of the modified SPM interferometer with acoustic stimuli at various sound pressure levels (SPLs). For the stimulus protocol, see **Supplementary Fig. 3b**. The frequency of the stimuli was 21 kHz. Five guinea pigs in total were assayed. Three conditions were examined in each animal: control (*red*), administration of salicylate crystals (*blue*), and the *post mortem* condition (*black*), as mentioned in the *main text*. The animal identification number is given in the *upper left corner* of each set of panels. The acquired data were subjected to the analysis described in **Fig. 4.**

Supplementary Table 1 Conditions of individual guinea pigs during experiments. The thresholds of the auditory brainstem response (ABR) were determined before (*Pre*: second row) and immediately after (*Post*: third row) measurements of cochlear-partition movements under control conditions in the guinea pigs assayed in **Figs. 1–4** and **Supplementary Figs. 4** and **5**. The results are displayed in the table. The animal identification numbers and threshold shifts are provided in the top and bottom rows, respectively.

Animal ID number(GP#)		494	528	537	545	548	235	257	258	396	398
ABR threshold (dB)	*Pre*	30	30	30	20	30	30	30	30	20	30
	Post	40	30	40	20	50	40	50	50	20	40
Threshold shift (dB)		10	0	10	0	20	10	20	20	0	10
