# Supplementary material for: Characterisation of the static offset in the travelling wave in the cochlear basal turn
Source: Pflugers Arch. 2020 Apr 22;472(5):625–35. doi: 10.1007/s00424-020-02373-6 (PMC7239825; doi:10.1007/s00424-020-02373-6)
Supplement: Supplementary file 2 — (PDF 594 kb). [file 424_2020_2373_MOESM2_ESM.pdf]

Characterisation of the static offset in the travelling wave in the cochlear basal turn

Pflügers Archiv - European Journal of Physiology

Takeru Ota[1,†], Fumiaki Nin[1,†,*], Samuel Choi[2,3], Shogo Muramatsu[3], Seishiro Sawamura[1], Genki Ogata[1], Mitsuo P. Sato[4], Katsumi Doi[4], Kentaro Doi[5], Tetsuro Tsuji[5,#], Satoyuki Kawano[2,5], Tobias Reichenbach[6], Hiroshi Hibino[1,2,*]

[1]Department of Molecular Physiology, Niigata University School of Medicine, Niigata 951-8510, Japan.

[2]AMED-CREST, AMED, Niigata 951-8510, Japan.

[3]Department of Electrical and Electronics Engineering, Niigata University, Niigata 950-2181, Japan.

[4]Department of Otolaryngology, Kindai University Faculty of Medicine, Osaka 589-8511, Japan.

[5]Department of Mechanical Science and Bioengineering, Graduate School of Engineering Science, Osaka University, Osaka 560-8531, Japan.

[6]Department of Bioengineering, Imperial College London, London SW7 2AZ, United Kingdom.

[†]These authors contributed equally to this work.

[#]Present address: Department of Advanced Mathematical Sciences, Graduate School of Informatics, Kyoto University, Kyoto 606-8501, Japan.

***Correspondence and material requests**: *H. Hibino*, Department of Molecular Physiology, Niigata University School of Medicine, 1-757 Asahimachi-dori, Chuo-ku, Niigata 951-8510, Japan. Email: hibinoh@med.niigata-u.ac.jp, Tel.: +81 25-227-2071, Fax: +81 25-227-0460; *F. Nin*, Department of Molecular Physiology, Niigata University School of Medicine, 1-757 Asahimachi-dori, Chuo-ku, Niigata 951-8510, Japan. Email: nin@med.niigata-u.ac.jp, Tel.: +81 25-227-2073, Fax: +81 25-227-0460.

Electronic Supplementary Material

Online Resource 2: Supplementary Methods

Modification of the SPM method

The laser interferometry used in this study was constructed by combining two different methods described elsewhere: the SPM interferometry that quantifies the offset [SM6], which is the motion magnitude of a statically displaced sample object, and homodyne interferometry that is modified to measure the vibration amplitude of an oscillating target [SM5]. The algorithm for the analysis of interference signals in our system was similar to that in synthetic heterodyne interferometry [SM4].

In both the SPM and homodyne interferometry mentioned above, the reference mirror was sinusoidally vibrated. When the interference signals are processed by fast Fourier transform, multiple components that originate from the motions of the mirror and sample are obtained. In SPM interferometry [SM6], the offset magnitude is calculated from the two components whose frequencies correspond to one and two-fold frequency of the mirror's movement. Analysis in homodyne interferometry extracts the component at the same frequency as the mirror's motion and the component at the frequency of the target's oscillation [SM5]. Although obviously the procedure of SPM interferometry is different from that of homodyne interferometry, these two methods were integrated and functionally coupled in the modified SPM interferometry we constructed as indicated below, in **Supplementary Text** (**Online Resource 3**), and in **Supplementary Fig. 1a–d** (**Online Resource 1**).

In the interferometer displayed in **Supplementary Fig. 1a**, if both the reference mirror and object sample are in a resting state, then $s(t)$, which is the intensity of an interference signal at time point t, is written as

$$s(t) = B\cos\left(\frac{4\pi}{\lambda}L_{res}\right), \qquad (\mathbf{1})$$

where B (≥ 0) and λ are the interference amplitude and the wavelength of the laser, respectively. The latter was 780 nm in this study. L_{res} is a difference between the following two values: L_M, the distance from beam splitter II to the reference mirror, and L_S, the distance from this splitter to the object. The original SPM method quantifies L_{res} [SM6]. In interferometry, the reference mirror is sinusoidally vibrated at certain parameters: amplitude a_r, frequency f_r, and initial phase θ_r. With this arrangement, **equation 1** can be expressed as

$$s(t) = B\cos\left[\frac{4\pi}{\lambda}\{a_r\cos(2\pi f_r t + \theta_r) + L_{res}\}\right]. \qquad (\mathbf{2})$$

L_M, which is involved in L_{res}, is equivalent to the centre or mean position of the oscillating mirror. Subsequently, $s(t)$ in **equation 2** is processed by fast Fourier transform to obtain a frequency domain signal at frequency f, $F(f)$. In the SPM method, two prominent signals emerge at f_r and $2f_r$ (**Supplementary Fig. 1c**). In this context, frequency domain signals at these two frequencies, $F(f_r)$ and $F(2f_r)$, which are referred to as the first- and second-order reference signals, respectively, are complex functions:

$$F(f_r) = BJ_1\left(\frac{4\pi}{\lambda}a_r\right)\sin\left(\frac{4\pi}{\lambda}L_{res}\right)e^{i\theta_r} \quad (\mathbf{3})$$

and

$$F(2f_r) = BJ_2\left(\frac{4\pi}{\lambda}a_r\right)\cos\left(\frac{4\pi}{\lambda}L_{res}\right)e^{2i\theta_r}. \quad (\mathbf{4})$$

$J_m\left(\frac{4\pi}{\lambda}a_r\right)$ denotes the mth-order Bessel function of the first kind, with $m = 0$, 1, or 2 in this study. Furthermore, **equations 3** and **4** are also rewritten as

$$F(f_r) = |F(f_r)| \exp\{i\Phi(f_r)\} \, (\mathbf{5})$$

and

$$F(2f_r) = |F(2f_r)|\exp\{i\Phi(2f_r)\}, (\mathbf{6})$$

where $\Phi(f)$ is the phase component of the frequency domain interference signal. $|F(f_r)|$ and $|F(2f_r)|$ correspond to the magnitudes of the signals at f_r and $2f_r$, respectively ($|F(45\,kHz)|$ and $|F(90\,kHz)|$ in **Supplementary Fig. 1c**). Here, the ratio of **equation 3** to **equation 4** is given by

$$\left|\frac{F(f_r)}{F(2f_r)}\right| = \left|\frac{J_1\left(\frac{4\pi}{\lambda}a_r\right)}{J_2\left(\frac{4\pi}{\lambda}a_r\right)}\tan\left(\frac{4\pi}{\lambda}L_{res}\right)\right|. (\mathbf{7})$$

This equation (**eq. 7**) yields L_{res}, the key parameter for the following processes.

Next, consider a situation where the cochlear partition, which is immersed in an extracellular solution, perilymph, is acoustically stimulated. The induced motions involve sinusoidal vibrations characterised by amplitude a_s, frequency f_s, and initial phase θ_s as well as offset ΔL. In each cycle of the measurement protocol depicted in **Supplementary Fig. 3b**, the end of the recording in a resting state and the onset of the recording with the acoustic stimulation were separated by an interval of 2 ms. In this condition, L_{stim}, which is the difference between L_M and L_S during the recording period with the stimulation, obeys the following equation:

$$L_{stim} = L_{res} + n_{ri}(\Delta L + \beta), (\mathbf{8})$$

where n_{ri} is a refractive index, which is 1.0 in air or 1.4 in water [SM7], and β represents the slow background displacement induced by breathing and/or heartbeat in a living animal. Of note, L_{res}, which is again a factor in a resting state, was determined in advance in every recording cycle (**Supplementary Fig. 3b**). On the basis of **equation 2**, the interference signal can be provided by the following relation [SM2]:

$$s(t) = B\cos\left[\frac{4\pi}{\lambda}\{a_r\cos(2\pi f_r t + \theta_r) + a_s\cos(2\pi f_s t + \theta_s) + L_{stim}\}\right]. (\mathbf{9})$$

The fast Fourier transform of **equation 9** results in two complex functions:

$$F(f_r) = BJ_1\left(\frac{4\pi}{\lambda}a_r\right)J_0\left(\frac{4\pi}{\lambda}a_s\right)\sin\left(\frac{4\pi}{\lambda}L_{stim}\right)e^{i\theta_r} \quad (\mathbf{10})$$

and

$$F(2f_r) = BJ_2\left(\frac{4\pi}{\lambda}a_r\right)J_0\left(\frac{4\pi}{\lambda}a_s\right)\cos\left(\frac{4\pi}{\lambda}L_{stim}\right)e^{2i\theta_r}. \quad \textbf{(11)}$$

These interference signals, $F(f_r)$ and $F(2f_r)$, can be described as the forms of right-hand terms in **equations 5** and **6**, respectively. In our analysis, a_s was quantified by conventional homodyne interferometry [SM1, SM3]; this process requires $|F(f_r)|$ and $|F(f_s)|$. As in **equations 5** and **6**, $|F(f_r)|$ and $|F(2f_r)|$ represent the magnitudes of frequency domain interference signals at f_r and $2f_r$, respectively. Therefore, the ratio of these values is given as

$$\left|\frac{F(f_r)}{F(2f_r)}\right| = \left|\frac{J_1\left(\frac{4\pi}{\lambda}a_r\right)}{J_2\left(\frac{4\pi}{\lambda}a_r\right)}\tan\left(\frac{4\pi}{\lambda}L_{stim}\right)\right|. \quad \textbf{(12)}$$

In **equations 7** and **12**, f_r, λ, and a_r are constant or determined in advance, so that the former and latter formulae yield $|L_{res}|$ and $|L_{stim}|$, respectively. Note the cases when L, which represents L_{res} or L_{stim}, falls within the range of either **condition 13** or **condition 14**:

$$\frac{\pi}{2} < \frac{4\pi}{\lambda}L < \pi: \sin\left(\frac{4\pi}{\lambda}L\right) > 0 \text{ and } \cos\left(\frac{4\pi}{\lambda}L\right) < 0 \quad \textbf{(13)}$$

or

$$\frac{3\pi}{2} < \frac{4\pi}{\lambda}L < 2\pi: \sin\left(\frac{4\pi}{\lambda}L\right) < 0 \text{ and } \cos\left(\frac{4\pi}{\lambda}L\right) > 0. \quad \textbf{(14)}$$

In either case, the sign of $|L_{stim}| - |L_{res}|$ is opposite to that of $n_{ri}(\Delta L + \beta)$, which represents the actual motion of the cochlear partition, although the absolute values of the two are identical. In other words, the direction of the calculated offset is inconsistent with that of the actual movement. Therefore, when our system, which monitored the interference signals in real time, detected either **condition 13** or **condition 14**, it multiplied the obtained $|L_{stim}| - |L_{res}|$ by –1.

In particular, either condition was determined by two phase components, $\Phi(f_r)$ and $\Phi(2f_r)$, which are obtained from **equations 3**, **4**, **10**, and **11**. In our experiments, a_r and a_s never exceeded 100 nm. Accordingly, in each of the four above-mentioned equations, the sign of $J_m\left(\frac{4\pi}{\lambda}a\right)$ was always positive. If the sign of the sine function was negative in both **equations 3** and **10**, then $\Phi(f_r)$ was expected to shift by π, i.e. by 180°, from θ_r in the process of derivation of $\Phi(f_r)$ from **equation 5**. Similarly, $\Phi(2f_r)$ obtained in **equation 6** was expected to shift by π from $2\theta_r$ when the cosine function in **equations 4** and **11** was negative. On the basis of these observations, the direction of the calculated offset was corrected, if necessary, in each recording cycle.

On the other hand, data were deleted in the following condition. *In vivo*, β, which is introduced in **equation 8**, sometimes reached 200 nm (noise duration: ~100 ms) [SM3] and greatly exceeded the offset measured in the cochlear partition (<10 nm; see **Figs. 2** and **3**). In this context, **equations 10** and **11**, which are related to the quantification of L_{stim}, need to be revised again. In general, the sign of either $\sin x$ or $\cos x$ reverses when the value of x is shifted by π/2, i.e. by 90°. This is the case in **equations 10** and **11** when β exceeds 69.6 nm (in water; see **eq. 8**). As a consequence, the sine function in **equations 3** and/or the cosine function in **equations 4** should be affected. Accordingly, the

change of the sign during measurement indicates an overlap of significant background displacement with the cochlear partition; the recordings in such a situation were automatically discarded by the software that we developed and were excluded from subsequent analyses. This algorithm, which is outlined in **Supplementary Fig. 1e** and **Supplementary Text** in **Online Resource 3**, allowed us to accurately detect the small amplitudes of <10 nm of acoustically induced sinusoidal vibrations and the offset in the cochlear partition.

Measurement of the ABR

Stainless-steel needle electrodes were subcutaneously inserted into the posterior region of the neck (non-inverting) and under the pinna (inverting). Tone-burst stimuli at 21 kHz were delivered to the ear. The duration of the stimulation was 3 ms and contained a rising phase of 1 ms and a falling phase of 1 ms. For one series of the measurements, the total duration including an interval was 60 ms. Individual signals evoked from the auditory tract were amplified 5,000-fold and processed with a bandpass filter (0.3–2 kHz) in an amplifier (EX-1; Dagan Corp., MN, USA). The digitised signals were averaged 1,000 times (LabVIEW™ 2013 Service Pack 1 32-bit; National Instruments, TX, USA). Intensity of the acoustic stimuli was changed in 10 dB steps until a hearing threshold was determined (0 dB = 0.02 mPa).

Supplementary References

SM1. Chen F, Choudhury N, Zheng J, Matthews S, Nutall AL, Jacques SL (2007) In vivo imaging and low-coherence interferometry of organ of Corti vibration. J Biomed Opt 12:021006. doi:10.1117/1.2717134

SM2. Choi S, Nin F, Ota T, Sato K, Muramatsu S, Hibino H (2019) In vivo tomographic visualization of intracochlear vibration using a supercontinuum multifrequency-swept optical coherence microscope. Biomedical Optics Express 10. doi:10.1364/boe.10.003317

SM3. Cooper NP (1999) An improved heterodyne laser interferometer for use in studies of cochlear mechanics. J Neurosci Methods 88:93-102. doi:10.1016/S0165-0270(99)00017-5

SM4. Galeti JH, Kitano C, Connelly MJ (2015) Improved synthetic-heterodyne Michelson interferometer vibrometer using phase and gain control feedback. Appl Optics 54:10418-10424. doi:10.1364/Ao.54.010418

SM5. Khanna SM (1986) Homodyne interferometer for basilar-membrane measurements. Hear Res 23:9-26. doi:10.1016/0378-5955(86)90172-3

SM6. Sasaki O, Okazaki H (1986) Sinusoidal phase modulating interferometry for surface profile measurement. Appl Optics 25:3137-3140. doi:10.1364/Ao.25.003137

SM7. Welzel J (2001) Optical coherence tomography in dermatology: a review. Skin Res Technol 7:1-9. doi:10.1034/j.1600-0846.2001.007001001.x
