# Supplementary material for: Characterisation of the static offset in the travelling wave in the cochlear basal turn
Source: Pflugers Arch. 2020 Apr 22;472(5):625–35. doi: 10.1007/s00424-020-02373-6 (PMC7239825; doi:10.1007/s00424-020-02373-6)
Supplement: Supplementary file 3 — (PDF 448 kb). [file 424_2020_2373_MOESM3_ESM.pdf]

Characterisation of the static offset in the travelling wave in the cochlear basal turn

Pflügers Archiv - European Journal of Physiology

Takeru Ota[1,†], Fumiaki Nin[1,†,*], Samuel Choi[2,3], Shogo Muramatsu[3], Seishiro Sawamura[1], Genki Ogata[1], Mitsuo P. Sato[4], Katsumi Doi[4], Kentaro Doi[5], Tetsuro Tsuji[5,#], Satoyuki Kawano[2,5], Tobias Reichenbach[6], Hiroshi Hibino[1,2,*]

[1]Department of Molecular Physiology, Niigata University School of Medicine, Niigata 951-8510, Japan.

[2]AMED-CREST, AMED, Niigata 951-8510, Japan.

[3]Department of Electrical and Electronics Engineering, Niigata University, Niigata 950-2181, Japan.

[4]Department of Otolaryngology, Kindai University Faculty of Medicine, Osaka 589-8511, Japan.

[5]Department of Mechanical Science and Bioengineering, Graduate School of Engineering Science, Osaka University, Osaka 560-8531, Japan.

[6]Department of Bioengineering, Imperial College London, London SW7 2AZ, United Kingdom.

[†]These authors contributed equally to this work.

[#]Present address: Department of Advanced Mathematical Sciences, Graduate School of Informatics, Kyoto University, Kyoto 606-8501, Japan.

***Correspondence and material requests**: *H. Hibino*, Department of Molecular Physiology, Niigata University School of Medicine, 1-757 Asahimachi-dori, Chuo-ku, Niigata 951-8510, Japan. Email: hibinoh@med.niigata-u.ac.jp, Tel.: +81 25-227-2071, Fax: +81 25-227-0460; *F. Nin*, Department of Molecular Physiology, Niigata University School of Medicine, 1-757 Asahimachi-dori, Chuo-ku, Niigata 951-8510, Japan. Email: nin@med.niigata-u.ac.jp, Tel.: +81 25-227-2073, Fax: +81 25-227-0460.

Electronic Supplementary Material

Online Resource 3: Supplementary Text

The measurement procedure and evaluation of modified sinusoidal phase modulation (SPM) interferometry

We developed the modified SPM interferometer as depicted in **Supplementary Fig. 1a** with a program for analysis of the interference signals. Prior to *in vivo* experiments, we evaluated the performance of the interferometry with a piezoelectric element (**Supplementary Fig. 1b–g**). This target was electrically stimulated multiple times; in a pilot experiment, each stimulus lasting 18 ms consisted of an AC voltage of 21 kHz, 0.28 V peak to peak, with a rising phase of 1 ms and a falling phase of 1 ms, as well as a DC offset voltage of 0.2 V, followed by an 82 ms interval, as shown in **Supplementary Fig. 1b**. This figure also illustrates a set of recorded raw time domain interference signals. The signals during time points 2–7 ms after the onset of the stimulus were analysed for measurement of the amplitude of the sinusoidal vibrations. A comparison of such signals with those observed for 5 ms immediately before the onset was intended for determination of the offset (**Supplementary Fig. 1b**; see **Materials and Methods**). This protocol, which is characterised by extremely brief data acquisition [ST2], stemmed from the expectation that *in vivo* measurement of the offset of the cochlear partition in live animals would be sensitive to low-frequency noises such as breathing and heartbeat. The 5 ms data recorded without and with the stimulation in every cycle were converted to the frequency domain signals by means of fast Fourier transform in real time. In this analysis, the first- and second-order reference signals elicited by vibrations of the reference mirror were visible in a resting state of the piezoelectric element (**Supplementary Fig. 1c**). Upon the stimulation at 21 kHz, an additional signal at the same frequency as the object's oscillations was detected with mild modification of the first- and second-order reference signals (**Supplementary Fig. 1d**). These three signal types (i.e. five signals in total) were necessary for characterisation of both the sinusoidal vibrations and the offset in the sample (see **Materials and Methods**).

Supplementary Fig. 1e depicts the workflow of an experiment. We first determined the limit of detection (LOD) with the unstimulated piezoelectric element. In this procedure, we recorded the interference signals for 5 ms with an interval of 95 ms and measured the magnitude of the frequency domain signal at 21 kHz, which mimicked the stimulus frequency used for acoustic stimuli in animal experiments (see **Supplementary Fig. 1c**). This cycle was repeated 80 times. The mean + 3 SDs of the 80 data points were calculated and served as the LOD. Next, the sample was challenged with multiple cycles of the stimulus protocol presented in **Supplementary Fig. 1b**. In every cycle, the following two indices were automatically examined in the absence and presence of the electrical stimulation: (1) intensities of the interference signals elicited by the motions of the reference mirror and sample, and (2) the signals' phase information that mirrors slow background motion of the sample. When the former exceeded the LOD (**Supplementary Fig. 1c** and **d**) and the latter satisfied the criterion that rules out significant contamination with background noise (see **Materials and Methods** for details), the dataset was assumed to be valid and was used to compile a dataset of the vibration amplitude and offset magnitude of the target. The computer software that we developed

continued the recording until it successfully acquired 80 datasets; the average of all the measurements for each of the two parameters was finally computed as a data point (**Supplementary Fig. 1e**). Of note, the modified SPM method can determine whether the sample's offset is directed upwards or downwards over time (see **Materials and Methods**).

In the experiments displayed in **Supplementary Fig. 1f**, the piezoelectric element was provided with the same AC voltage as that used in **Supplementary Fig. 1b** and simultaneously a DC offset voltage of 0, 0.025, 0.05, 0.075, 0.1, or 0.25 V. A strain gauge was attached to the element to monitor the length change. Overall, the values of the offset detected by the modified SPM interferometer were consistent with those of the mean length change measured by the gauge within the range less than 18.6 nm (**Supplementary Fig. 1f**; coefficient of correlation, $r^2 = 0.9977$). A Bland–Altman plot indicated a small bias: 0.12 ± 0.53 nm (i.e. the mean difference between the values obtained with the two methods ± SD; **Supplementary Fig. 1g**). All the data points fell within the range of 95% confidence: −0.92 to 1.16 nm. These observations confirmed the quantitativity of the method that we developed for offset detection.

Acquisition of data for tuning curves to verify the experimental methods

To verify our animal preparation and experimental approach, we quantified sinusoidal vibrations of a cochlear partition while stimulating a guinea pig with tone-burst sounds of different combinations of frequencies (10 and 13–30 kHz spaced by 1 kHz) and intensities (40–90 dB SPL; 10 dB step; **Fig. 1c**). The duration of stimulation for a measurement was set to 10 s in total (i.e. 500 ms stimulus × 20 times; **Supplementary Fig. 3a**) in order to roughly compare the acquired data with the literature data [ST3]. In this context, the LOD was determined beforehand with frequency domain signals that were recorded over a period of 10 s from the cochlear partition in a resting state. When the magnitude of the stimulation was ≥40 dB SPL, the object's interference signals exceeded the LOD [ST4]. At the stimulus frequencies of 18–30 kHz, the response of the partition was highly tuned (**Fig. 1c**). At sound pressure 40 dB, the vibration amplitude ranged from 0.19 to 0.35 nm and sharply peaked at 23 kHz. The peak was observed at 50 dB stimuli of the same frequency but shifted to a lower frequency of 21 kHz at 60 dB SPL [ST1]. Compressive nonlinearity was obvious from sensitivity, which was calculated as the ratio of vibration amplitude to stimulus intensity (**Fig. 1d**). Among various frequencies, the 23 kHz stimuli evoked the largest difference (28 dB) in the sensitivity between 40 and 90 dB SPL. These properties of the tuning curve are similar to those described elsewhere [ST1, ST4].

Supplementary References

ST1. Cooper NP, Rhode WS (1997) Mechanical responses to two-tone distortion products in the apical and basal turns of the mammalian cochlea. J Neurophysiol 78:261-270. doi:10.1152/jn.1997.78.1.261

ST2. Cooper NP, Vavakou A, van der Heijden M (2018) Vibration hotspots reveal longitudinal funneling of sound-evoked motion in the

mammalian cochlea. Nat Commun 9:3054. doi:10.1038/s41467-018-05483-z

ST3. Lukashkin AN, Bashtanov ME, Russell IJ (2005) A self-mixing laser-diode interferometer for measuring basilar membrane vibrations without opening the cochlea. J Neurosci Methods 148:122-129. doi:10.1016/j.jneumeth.2005.04.014

ST4. Zha D, Chen F, Ramamoorthy S, Fridberger A, Choudhury N, Jacques SL, Wang RK, Nuttall AL (2012) In vivo outer hair cell length changes expose the active process in the cochlea. PLoS One 7:e32757. doi:10.1371/journal.pone.0032757
